# Financing the cultural and creative industries through crowdfunding: the role of national cultural dimensions and policies

**DOI:** 10.1007/s10824-022-09452-9

**Published:** 2022-06-18

**Authors:** Antonella Francesca Cicchiello, Serena Gallo, Stefano Monferrà

**Affiliations:** 1 Dipartimento di Studi Scienze Economiche e Sociali, Università Cattolica del Sacro Cuore, Piacenza, Italy; 2 Dipartimento di studi Aziendali e Quantitativi, Università degli Studi di Napoli Parthenope, Naples, Italy; 3 Dipartimento di Scienze Economiche e Sociali, Università Cattolica del Sacro Cuore, Piacenza, Italy

**Keywords:** Reward-based crowdfunding, Cultural and creative industries, Cultural policy, European countries, G20, G23, Z1, Z18

## Abstract

The trend towards digitalisation and technological innovation has reshaped the cultural and creative industries (CCIs) by changing the existing funding models and structures. The aim of this article is to explore the impact of cultural dimensions and policies on the adoption of reward-based crowdfunding as a new form of finance for firms in the CCIs in 12 different European countries during the 2015–2019 period. Our results show that national cultural dimensions and policies significantly affect the demand for cultural and creative crowdfunding. Specifically, the adoption of crowdfunding is broader in individualistic countries and in societies characterised by higher uncertainty avoidance, indulgence, short-term orientation, and lower levels of discrimination between genders. Furthermore, we find that the liberal welfare state model, characterised by limited government interference, market orientation, privatisation and a focus on self-responsibility, and the Southern European welfare model, based on a weak and inefficient state, increase the adoption of crowdfunding in the CCIs. The presence of a central ministry with cultural competence also increases the adoption of crowdfunding in the CCIs. Our findings show a U-shaped relationship between European grants and the demand for crowdfunding, mainly driven by a high or low European involvement within these sectors. We also identify a moderation effect of EU grants on the relationship between cultural dimensions and crowdfunding adoption, suggesting that the magnitude of this relationship depends on the amount of EU grants awarded in a specific country. As a robustness check, we run a set of Poisson regressions with correlated random effects (CREs), confirming our main results.

## Introduction

New digital technologies have radically changed the economy, generating consequences in all economic sectors and starting what is called the Fourth Industrial Revolution (Industry 4.0) (Morrar et al., 2017). The trend towards digitalisation and technological innovation has reshaped the cultural and creative industries (CCIs), especially the music and film industries, by changing the existing arts funding models and structures (Tosatto et al., 2019). The reconfiguration of the financial services industry following the new financial technologies, known as FinTech, is helping bridge the funding gap that particularly affects firms in the CCIs, which are disadvantaged in accessing traditional sources of funds, such as banks, venture capitalists and business angels (Lazzaro, 2017). On the one hand, the emergence of new online financial platforms (such as crowdfunding platforms), with lower transaction fees and new techniques and sources of information for assessing credit risk, has helped promote access to credit for micro firms^1^ and SMEs in CCIs. On the other hand, new payment solutions and digital tools not only promote the digitisation of transactions (Crupi et al., 2020) but also create new options for resolving information asymmetries—by making transaction history and digital footprints available to assess credit risks—with positive consequences for SMEs and microbusinesses operating in those industries in terms of the opportunity to obtain financial support. Belonging to the FinTech revolution, crowdfunding (CF) is a comprehensive term used to describe a new form of funding projects, companies or ideas by raising many small amounts of capital from a large number of people, typically via online platforms (Ahlers et al., 2015; Cicchiello, 2020). This novel form of financial intermediation makes it easier for those seeking funding (whether individuals or companies) to reach a high number of potential investors, who receive some form of physical or moral reward in proportion to the invested funds (for a detailed description, see Belleflamme et al., 2014). Beyond traditional forms of financing, crowdfunding has recently emerged as a new player in entrepreneurial finance (Block et al., 2018; Cicchiello & Leone, 2020), significantly reducing the funding gap for cultural and creative firms that make an important contribution in Europe in economic and social terms (Hutter & Throsby, 2008; Klamer, 1996). These firms stimulate innovation throughout the economic sphere and contribute to generating a positive social impact in numerous other areas, such as well-being and health, education, inclusion and urban regeneration (Konrad, 2013). Since its inception in 2006, various models of crowdfunding have been created, depending on the way in which investors are recompensed.^2^

Recent literature has recognised that crowdfunding (notably in reward-based form) has the potential to democratise the process of funding artistic and cultural ventures (Brabham, 2017). The democratising force of crowdfunding platforms in financing firms within the cultural and creative industries is due to their role in facilitating the interaction between founders and a multitude of nonprofessional small investors through the use of the internet, without standard financial intermediaries (Mollick, 2014). While traditional funding channels are limited to a small group of expert critics (such as venture capitalists and grant-making bodies) who play crucial roles in allocating artistic and culture funding (Woronkowicz et al., 2012), crowdfunding provides those seeking funding with the opportunity to raise funds from diversified sets of “crowd investors” in a digital environment, replacing the judgement of a few experts. Early empirical observations from Europe and the USA reveal the positive outcomes of crowdfunding in CCIs (Boeuf et al., 2014; Mollick & Nanda, 2016). Cultural and creative projects may have more funding opportunities when targeting crowdfunding, especially in industries (such as the theatre and film industry) where the crowds are end-users (Mollick & Nanda, 2016). The aim of this article is to explore the impact of national cultural dimensions and policies on the adoption of reward-based crowdfunding as a new form of finance for European firms in CCIs. Cultural policies are a valuable tool for laying the foundations for long-term growth and innovation in CCIs. They can help support and expand artists and firms in the CCIs by providing the liquidity they need and relieving some of the financial pressure caused by the lack of funding from the private sector. National cultural policies to support CCIs can contribute to protecting cultural and creative workers and businesses from economic crises, such as the recent crisis induced by the COVID-19 pandemic. Although these policies may appear uniform, they have different impacts based on the scale of the creative and cultural sectors across different local contexts. For this reason, it is important to study cultural policies in relation to the national cultural dimensions that collectively portray the impact of the culture ingrained in society on the values of the members of that society.

Given the importance of CCIs to the European economy and the difficulties currently faced by the industry in finding new forms of financial sustainability (Collins, 2018), our analysis is particularly important and timely. Since the diffusion of crowdfunding among countries differs consistently based on differences in national institutional environments (Di Pietro & Butticè, 2020), it is crucial to study the association among cultural dimensions, cultural policies, and country-level crowdfunding activity. To the best of our knowledge, this is the first study to econometrically investigate this association in the cultural and creative industries. To do this, an original dataset has been collated and utilised, considering the universe of creative and cultural projects launched on Kickstarter—the largest and dominant reward-based crowdfunding platform based in the UK—in 14 European countries^3^ over the period 2015–2019. For each project, released data are collected on the founders’ country and the categories in which the projects are listed. Country-level information is also collected from various publicly available databases to create variables related to European cultural policy. The variables relating to cultural dimensions are extracted from the Hofstede dataset (Hofstede, 1991, 2001). Specifically, we include (1) the dimension “*Individualism Versus Collectivism”,* measuring the degree to which people give priority to their personal goals over in-group goals; (2) the “*Uncertainty Avoidance Index”* (high *versus* low), measuring how uncomfortable the members of a society feel regarding uncertainty and ambiguity; (3) the “*Power Distance Index”* (high *versus* low), expressing the degree to which the less powerful members of a society accept and expect that power is distributed unequally; (4) the dimension “*Masculinity versus Femininity”,* referring to what values are considered more important in a society; (5) the dimension “*Indulgence versus Restraint”,* measuring the importance a society attributes to allowing or encouraging freedom, leisure, happiness, and free gratification; and (6) the dimension “*Long- versus Short-Term Orientation”,* considering the extent of a society’s time horizon. Finally, these data are integrated with the World Bank’s indicators; with data on companies active in the cultural and creative sectors extracted from the Eurostat database; and with information on banking market concentration and national cultural policy from the European Central Bank (ECB) and the European Commission, respectively.

As discussed in detail below, our study finds that national cultural dimensions and policies significantly affect the demand for cultural and creative crowdfunding. Specifically, our analysis shows that the adoption of reward-based crowdfunding for cultural and creative activities is broader in individualistic countries and in societies characterised by higher uncertainty avoidance, indulgence and short-term orientation. However, in countries characterised by higher power distance and masculinity, cultural and creative firms are less prone to using crowdfunding. Our study also highlights that the adoption of reward crowdfunding among creative and cultural firms is larger in countries with high patent applications, e-commerce reliability, a favourable legal environment and a lower concentration of the banking market. Finally, among the various welfare state models analysed (such as liberal, Central European, Nordic and Southern), we find that the liberal welfare state model, characterised by limited government interference, market orientation, privatisation and a focus on self-responsibility, and the Southern European welfare model, based on a weak and inefficient state, increase the adoption of crowdfunding in CCIs. Furthermore, we find evidence that the presence of a central ministry with cultural competence positively influences the adoption of crowdfunding. Our findings show a nonlinear relationship between European grants awarded under the *Creative Europe Programme* and the demand for crowdfunding. Specifically, when EU grants are higher than a critical value, they have a restraining effect on the demand for crowdfunding by firms in CCIs. We also identify a moderation effect of EU grants on the relationship between cultural dimensions and crowdfunding, suggesting that the magnitude of their relationship depends on the amount of EU grants. However, the moderation effect is partially confirmed in our empirical models since only three interaction terms between EU grants and cultural dimensions (e.g. Individualism, Power distance and Indulgence) have significant coefficients. This paper contributes to the growing literature on cultural and creative entrepreneurship (CCE), which is developing as a field of research per se (Sinapi, 2020). Here, one particular research gap that needs to be explored is the impact of cultural dimensions and policies on cultural and creative entrepreneurs’ possibility of raising capital in the reward crowdfunding market. Overall, given the democratisation potential of crowdfunding, it could facilitate access to capital for cultural and creative entrepreneurs in Europe, thus fostering CCI growth.

A second gap in the literature concerns our limited understanding of the country-level determinants of crowdfunding in CCIs. European countries differ in terms of the geographical spread of cultural amenities and activities, cultural participation and specialisations in the cultural and creative sectors (European Commission, 2018a), as well as in terms of crowdfunding volumes (Ziegler et al., 2019). Therefore, it is important to analyse how national-level characteristics, such as the availability of public funding, cultural spending, nationalistic tendencies, regulatory environment, cultural dimensions and policies, may all impact the demand for cultural and creative crowdfunding in different environments. While some prior studies have used context to explore at a microlevel what makes a specific campaign successful (e.g. Josefy et al., 2017), few studies examine the degree to which the cultural context may influence at a macrolevel the adoption of crowdfunding itself in different environments. Therefore, in this paper, we made a pioneering attempt to unravel the linkage between cultural dimensions, cultural policies and crowdfunding adoption in the cultural and creative industries. Finally, our study contributes to the field of cultural economics and cultural policy research by investigating for the first time the association among cultural dimensions, cultural policies and Europe’s crowdfunding activity in CCIs.

## Theoretical background

### Funding challenges in the CCIs

Due to its multidimensional nature and the existence of various approaches in different jurisdictions, there is no universally accepted definition of CCIs in the literature (Peltoniemi, 2015; Potts et al., 2008).

This study adopts the perspective of the Creative Europe Programme,^4^ which considers the cultural and creative industries (recently also termed “cultural and creative sectors”) as follows: “*all sectors whose activities are based on cultural values and/or artistic and other creative expressions, whether those activities are market- or nonmarket-oriented, whatever the type of structure that carries them out, and irrespective of how that structure is financed”*. Those sectors include, inter alia, architecture, archives, libraries and museums, artistic crafts, audio-visual (including film, television, video games and multimedia), tangible and intangible cultural heritage, design, festivals, music, literature, performing arts, publishing, radio and visual arts. The literature on entrepreneurial finance recognises that European firms operating in CCIs face difficulties and barriers in raising finance (Boeuf et al., 2014; Collins, 2018; Hackett et al., 2000; Landoni et al., 2020; Lazzaro, 2017). Although cultural and creative firms in Europe contribute 4.4% (approximately €509 billion) of the GDP and 7.5% (12 million full-time jobs) of the total workforce,^5^ it is estimated that the financing gap for European firms operating in CCIs was somewhere between €8 and €13 billion over the period 2014–2020 (European Commission, 2018b). On the demand side, this financing gap can be partly explained by the aversion of individuals and firms operating in CCIs to the use of external funding, such as loans and direct investments (Poettschacher, 2005; Sigurdardottir & Candi, 2019). Previous studies have confirmed that creatives may not feel comfortable asking for money (Lin & Phillips, 2017); thus, they prefer to finance their business through strategic alliances (Gundolf et al., 2018). This emphasis on financial independence can be linked to the need to control artistic content—creative entrepreneurs generally crave artistic freedom (Peltoniemi, 2015; Sigurdardottir & Candi, 2019)—and the presence of barriers based on personal characteristics, cultural background and the entrepreneurial environment (Sundbo, 2011). The entrepreneurs in these firms typically have a creative background, without any previous financial knowledge or managerial capabilities and experience (Sundbo, 2011). Furthermore, conflicts between artistically motivated creatives and financially motivated managers are common in creative or cultural production (Eikhof & Haunschild, 2007). The need to satisfy consumers’ continually evolving tastes often clashes with the inclinations of creatives, who may refuse strategies proposed by managers if they do not fit with their artistic integrity and meet their required quality standards (Tschang, 2007; Wei, 2012). Managers involved with the creation, production, marketing and distribution of cultural and creative products and services have the arduous task of reconciling the economics of mass entertainment with the individual inspiration of creators, which is ultimately at the root of creating value in the cultural and creative industries (Lampel et al., 2000). On the supply side, the limited access of firms in CCIs to external funding may be related to the existence of bias and stereotypes by investors who distrust artistic and creative producers, especially in some newer and investment-heavy creative sectors, such as video games and digital animation industries (Cunningham et al., 2008; Hackett et al., 2000; O’Dair & Owen, 2019; Ryan, 2010). The CCIs are composed largely of microenterprises (less than 10 employees), non-profit organisations and creative professionals characterised by a high degree of uncertainty resulting from the nature of the product—ideas and information whose value depends upon a subjective judgement at the point of consumption—and the nature of creative processes, which do not follow a linear route (there is no sure correlation between input and output) (Caves, 2000). For most professionals and entrepreneurs in CCIs, economic growth or profit does not represent the main objective of their activities but rather a useful tool to pursue their intrinsic goal, i.e. the fulfilment of their creative process. In their role of integrating “art and commerce” (Caves, 2000), companies in CCIs rely on intellectual capital and intangible assets, such as highly specific forms of skills and competences, networks of social relationships, or reputation and credit in specific creative communities (Borin & Donato, 2015). The intangible nature of these assets makes it difficult for companies to access traditional forms of financing, as lenders may be reluctant to provide credit due to difficulties in valuing these assets. Funding challenges for businesses in CCIs are also linked to the unclear relationship between cultural capital and economic capital. Indeed, returns on investment in culture are difficult to quantify, whether in terms of positive externalities for the public good or actual profits for shareholders (Fraser & Lomax, 2011). These factors make firms in CCIs an extremely risky proposition for external investors (Bilton, 1999). The lack of private financing hinders the growth of CCIs; firms tend to remain small and often fail, and many creative projects remain within the realm of ideas without having the chance to be developed (Collins, 2018; Landoni et al., 2020). Furthermore, barriers to financing creative industries in Europe are exacerbated by the inadequacy and instability of public funding for the arts and the lack of support available from the government and other institutions (Marchegiani, 2018). In this context, alternative and innovative sources of finance, such as crowdfunding, have the potential to bridge the funding gap that prevents the cultural and creative industries from growing, creating more jobs and stimulating economic and social renaissance across Europe (De Voldere & Zeqo, 2017).

### Crowdfunding in the CCIs

Given the shrinking public and private funding in CCIs globally, crowdfunding has become an increasingly popular funding channel for the arts and culture (Lazzaro & Noonan, 2020). Over the past few years, an increasing number of cultural organisations and creatives have appealed to the disparate and dispersed community of internet users for funding. According to De Voldere and Zeqo (2017), more than 75,000 crowdfunding campaigns have been launched since 2013 in CCIs, with some €247 million raised by the crowd. The UK is the largest crowdfunding market in the CCIs, with 36% of campaigns and 41% of transaction volume over the period 2013–2016, followed by France (30% of campaigns and 22% of transaction volume).

**Fig. 1:**
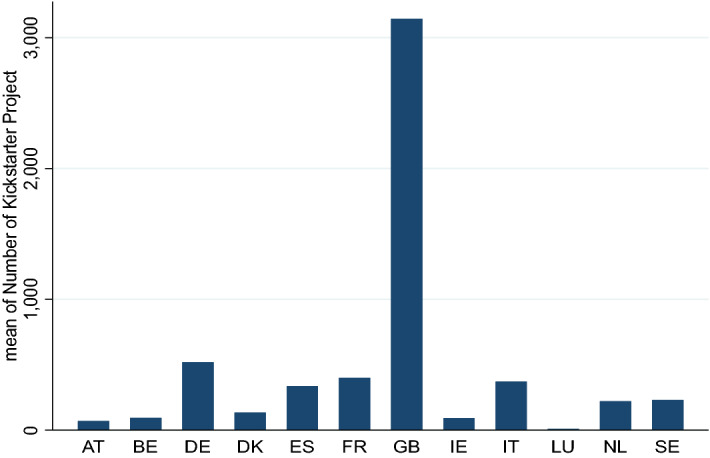
Number of cultural and creative projects on Kickstarter by EU countries. *Source*: Authors’ elaboration based on Kickstarter data. *Note* This figure reports the cultural and creative projects on Kickstarter by 12 EU countries involved in our dataset over the period 2015–2019

Crowdfunding in the CCIs is typically run in reward-based form (Tosatto et al., 2019); thus, funders receive a nonmonetary reward (either symbolic or in-kind) or products based on the amount of money they bring to the project (Mollick, 2014). According to the 4th annual European Alternative Finance Industry Report (Ziegler et al., 2019), the reward-based crowdfunding market reached €159 million in 2017, with 35% of volume going to businesses in the CCIs (including arts, music and design sectors). Although there are over 600 platforms in Europe, almost half of the CCI campaigns (47%) are hosted on the global US-based platforms Kickstarter and Indiegogo (Voldere & Zeqo, 2017). Kickstarter remains the largest and dominant reward-based crowdfunding platform in Europe focusing on cultural and creative projects. As shown in Fig. 1, there is a strong variance in the number of crowdfunding projects across European countries. The UK, on average, presents the highest number of projects on Kickstarter in cultural and creative categories, followed by Germany, France and Italy.

Given the rapid growth of crowdfunding in the CCI (Tosatto et al., 2019), increasing attention has arisen among practitioners, policy makers and researchers alike. The existing literature on crowdfunding in CCI mainly focuses on the determinants of crowdfunding campaigns’ success (Rahimli & See-To, 2017; Tosatto et al., 2019). In one of the first exploratory studies on the Kickstarter platform, Mollick (2014) provides interesting statistics of projects in the CCIs. In particular, the author finds that more campaigns fail than succeed in their fundraising and that the majority succeed by only small amounts; music campaigns are more likely to be successfully funded but also have the lowest average goal amount. Furthermore, the presence of a pitch video, the founder’s social media usage, number of updates and general indicators of campaign quality are determining factors for the success of campaigns. Similarly, Kuppuswamy and Bayus (2017, 2018) examine the funding dynamics of campaigns on Kickstarter and reveal that funders are more likely to contribute in the first or last weeks of the campaign. Furthermore, the authors identify the so-called Kickstarter effect, whereby the probability of success increases as the funding goal approaches. These results are consistent across all campaign categories, including KICs. In a sample of 875 successful theatre projects financed through Kickstarter in 2011, Boeuf et al. (2014) show that the offer of symbolic rewards, such as public acknowledgement of the donor, is positively associated with the amount of capital raised for a project and its subsequent success, but only when no material rewards are offered. By analysing 100 creative crowdfunding campaigns within the film and video category from Kickstarter, Hobbs et al. (2016) find that pitch quality and updates, as well as network management, are key drivers of crowdfunding success. In contrast to the idea that crowdfunding reduces the inhibitory effect of the distance between donors and entrepreneurs, Mendes-Da-Silva et al. (2016) reveal a significantly negative association between distance and the propensity of donors to back music production projects on the Brazilian crowdfunding platform Catarse. Analysing 447 crowdfunding campaigns for video games, Cha (2017) suggests that human capital in terms of prior professional experience, geography, media choice, and the intensity of media use (measured by the number of images, videos, and graphics included in the campaign) influence the campaigns’ success. Similarly, Bao and Huang (2017) find that external factors, such as reward support, impression management and social capital, have positive effects on the success of film and video and publishing projects. Another study by Bi et al. (2017) reveals that project quality signals captured by campaign text length and the spread of e-word-of-mouth in terms of “likes”, comments, and shares have significant positive effects on funder investment decisions for entertainment and art projects. In their study on crowdfunding for art projects in the UK, Lin and Phillips (2017) confirm that the presence of a clear and transparent video disclosing the content and uniqueness of projects, as well as an appropriate funding target and geographical proximity to the artists, positively influence the success of the campaigns. In the context of equity-based crowdfunding, Vrontis et al. (2020) reveal that campaigns’ success rate in the Italian market is positively related to intellectual capital and significantly related to the number of connections the platforms have. Del Sarto and Magni (2018) find evidence that the dynamic capabilities of firms positively influence the implementation of a successful equity crowdfunding campaign. Zheng et al. (2020) show that knowledge management promotes knowledge-driven business model innovation through equity investment. According to Galuszka and Bystrov (2014), one of the most important factors for the success of campaigns on the Polish music equity crowdfunding platform MegaTotal is the involvement of a significant number of backers who make repeated contributions to a project. Engagement in communication with potential backers and offering bonuses to contributors may help a project succeed.

Another stream of literature analyses the reasons behind the use of crowdfunding in CCI, assuming that creators and funders could be driven by more than the possibility of securing funding and consuming products and experiences, respectively (Gerber et al., 2012; Huang, 2020). From a funder perspective, for example, Gerber et al. (2012) find that participation in crowdfunding in cultural industries is motivated not only by the opportunity to obtain some product (e.g. a DVD or CD) in return but also by the feeling of connectedness to a community with similar interests and ideals. From the creators’ point of view, the authors find that crowdfunding represents more than a funding channel. Indeed, participation in crowdfunding allows creators to build long-term social interactions and obtain feedback on their ideas. According to Aitamurto (2011), the primary motivation for donating in the crowdfunded journalistic process is to contribute to the common good and social change. Using a survey of crowdfunding project founders in CCIs, Davidson and Poor (2015) explore the relationship between certain social and psychological characteristics of project backers and attitudes towards the use of crowdfunding. According to the authors, crowdfunding appears to advantage producers with particular personality structures, such as extraversion, while disadvantaging others. Based on a survey addressing supporters of reward-based crowdfunding campaigns in the field of video game development, Steigenberger (2017) provides empirical evidence that supporters are driven not exclusively by a purchasing motive but also by altruistic and involvement motives. Marchegiani (2018) confirms that the willingness to contribute to a crowdfunding campaign is positively associated with the expectations of contributing to the creative process and establishing a relationship with the crowdfundee(s). In a very recent study, Huang (2020) examines the motivations driving film fans’ willingness to sponsor projects published on film crowdfunding platforms. The results of 505 valid reports reveal that film fans’ willingness is influenced by the perceived value of nonmaterial feedback rather than the perceived value of material feedback. The author also finds evidence that film fans’ perceived risks have significant mediating effects on the relationship between the willingness to contribute to film projects and incentives for film crowdfunding; film fans’ perceived convenience did not have a significant mediating effect. Other studies contribute to understanding the effects of crowdfunding on the value creation process in CCIs (Jose Planells, 2017; Nucciarelli et al., 2017). Aitamurto (2011), for example, discusses the relationship between crowdfunding and journalism in the value creation process. The author states that crowdfunding, by requiring journalists to renegotiate their role and professional identity, creates a new sense of responsibility of journalists towards donors. Analysing the digital game industry, Nucciarelli et al. (2017) show that in addition to fundraising, the use of crowdfunding has the advantage of unifying the channels that bring capital, technology and knowledge of the market from the crowd into the game. Cameron (2016) defines crowdfunding as a benefit to the music industry, as it is able to compensate for the loss of revenue caused by copyright infringement. According to the author, the negative aspects of crowdfunding, such as scams and fraud (Cumming et al., 2020a, b), tend to be limited in the music industry, as those providing funds are able to determine the authenticity of a project. In his study, Jose Planells (2017) describes crowdfunding not only as a unique opportunity but also as a real revolution for the video game industry. According to the author, crowdfunding opposes the current hegemonic production model based on the rigid and traditional policies of publishers who, being exclusively focused on the constant maximisation of profit, are unable to perceive the creative, artistic or entertainment value of the gaming industry. Through a bottom-up participatory culture, crowdfunding transforms consumers into prosumer investors eager for new play experiences, allowing game developers to cut out the middleman (i.e. the publisher) and establish a direct connection with prosumers. Galuszka and Brzozowska (2017) underline the democratising influence of crowdfunding on the music market and its power to enable artists to enter the music market without the intermediation of traditional record labels. However, the authors show that the democratising influence of crowdfunding in the music market is limited by the fact that neither platforms nor contributors have the power, connections and know-how of traditional record labels. Similarly, Gamble et al. (2017) state that crowdfunding is changing the business models of the music industry in terms of creativity, strength and relationships with artists. Looking at the benefits of the crowdfunding financial model, major record labels are currently considering a more user-centric financial model as an innovation strategy. Loriguillo-López (2017) confirms that crowdfunding is transforming the precarious Japanese commercial animation industry, allowing its development among potential sponsors, especially among fans based outside Japan. Despite their individual limitations, the aforementioned studies collectively explain why crowdfunding has started to become an important source of finance for businesses in the CCI (Collins, 2018).

### Cultural characteristics and crowdfunding performance

Prior studies have shown that formal institutions and (informal) cultural values may influence the levels and types of entrepreneurial activity in different countries by shaping both environmental conditions and individual orientation for entrepreneurship (Mueller & Thomas, 2001). Formal institutions, as a set of political, economic and contractual rules that regulate individual behaviour and shape human interaction, can influence access to funding and other resources (e.g. Cumming & Zhang, 2019; Li & Zahra, 2012), creating environmentally favourable (or unfavourable) conditions for new venture creation and other entrepreneurial activities. For example, among the many formal institutional characteristics, the level of regulatory complexity, in terms of ease of doing business, protections of minority shareholders rights, procedures related to starting a new business and legal system efficiency, can act as a promoter or inhibitor of entrepreneurship (Busenitz et al., 2000). The effects of formal institutions depend on the informal cultural settings that embody the set of values, attitudes, beliefs and norms of behaviour that are socially transmitted and constitute the national cultural heritage (North, 1990). Formal institutions are embedded in cultural settings because political, economic and contractual rules are all connected to peoples’ conceptions of how things should be done. As a result, the same formal institutions that exist in societies with different cultural values can produce different economic outcomes (North, 1990), affecting the propensity for financial investors (e.g. angel investors, venture capitalists) to fuel innovation and the development of new ventures (Li & Zahra, 2012) as well as the propensity of individuals to cultivate the mind and character of the potential entrepreneur (Busenitz et al., 2000). This means that culture can affect the potential for entrepreneurship, generating differences across national and regional boundaries, as some cultures may be more conducive to entrepreneurship than others (Lee & Peterson, 2000). Using the six cultural dimensions proposed by Hofstede et al. (2004) (i.e. individual tendency to avoid uncertainty, individualism attitude, power distance, long-term orientation, indulgence, and masculinity), existing studies have shown that informal cultural characteristics influence entrepreneurial finance and entrepreneurial activity across countries (e.g. Li & Zahra, 2012; Mueller & Thomas, 2001). According to Mueller and Thomas (2001), for example, an entrepreneurial orientation, defined as an internal locus of control combined with innovativeness, is more likely in individualistic, low uncertainty avoidance cultures than in collectivistic, high uncertainty avoidance cultures. In their study, Li and Zahra (2012) show that the variation of venture capital activity across countries is attributed to the different levels of formal institutional development and that this effect is weaker in uncertainty-avoiding and collectivist societies.

Recognising crowdfunding as a new player in entrepreneurial finance (Block et al., 2018; Bruton et al., 2015), scholars have started to investigate the role of formal institutions and (informal) cultural values on the demand for and supply of crowdfunding across countries. Using data from China and the USA, Zheng et al. (2014) carry out one of the first comparative empirical studies on the differences in crowdfunding across cultures. The authors find evidence that the national culture moderates the effect of social capital on crowdfunding performance, with a greater influence on the Chinese collectivist society in which people place more importance on personal relationships. According to Burtch et al. (2014), crowdfunders prefer culturally similar and geographically proximate fund seekers. Josefy et al. (2017) investigate the role of community culture on the success of crowdfunding campaigns to “save the local theater”. According to the authors, communities with cultures in keeping with the nature of the project are most likely to fund the relevant venture. By applying Hofstede’s (2004) cultural dimensions, Cho and Kim (2017) demonstrate how cultural distinctiveness is portrayed in crowdfunding projects. Di Pietro and Butticè (2020) analyse the influence of formal institutions (i.e. the level of regulatory complexity and financial market development) and informal institutions (i.e. the six cultural dimensions of Hofstede et al. (2004)) on the development of different crowdfunding typologies. The authors find evidence that the crowdfunding market is larger in countries with a business-friendly legal environment and well-developed financial markets. Regardless of the typologies, crowdfunding is more widespread in individualistic societies. Lending- and equity-based crowdfunding is prevalent in long-term oriented societies, while countries characterised by higher uncertainty avoidance register higher lending-based crowdfunding activity. Although prior studies have analysed the impact of formal and informal institutions on crowdfunding development, none of them have focused on CCIs (Rykkja et al., 2020). In this paper, we fill this gap by investigating for the first time the linkage between cultural dimensions and Europe’s crowdfunding activity in CCIs. Based on the above, we explore the following hypothesis:

#### **H1:**

 National cultural dimensions affect the adoption of reward-based crowdfunding in CCIs.

Cultural sectors play an essential role as resources for attracting investment and tourists or for encouraging the export of products. Thus, it is not surprising that for many countries, culture has become an increasingly central political field (Rubio Arostegui & Rius-Ulldemolins, 2020). Cultural policies can be considered the totality of a government’s activities “with respect to the arts (including the for-profit cultural industries), the humanities, and the heritage” (Schuster, 2003). These policies involve all governmental strategies and activities aimed at promoting “the production, dissemination, marketing, and consumption of the arts”. The formulation of cultural policies is strongly conditioned by the historical, social, economic and political context in which they develop, which differs from country to country. In Europe, for example, cultural policies were the domain of public interventions and had a multitude of definitions until the start of the nineties, when convergence toward a relatively common definition began. The great diversity in the way cultural policies are defined, developed and implemented makes comparison among them difficult. Over the years, many authors have attempted to analyse the different structures, characteristics and modes of functioning in cultural policies, with the aim of establishing a classification that would allow a comparison on a global level. Among them, based on the typology of the welfare state framework, Zimmer and Toepler (1996) have identified three models: (i) the liberal model—a model characterised by weak state interventionism, market-orientation, privatisation and focus on self-responsibility (different submodels from Great Britain and the USA belong in this category), (ii) the Central European model—characterised by strong state-level support for high culture (the paradigmatic example of this is France), and (iii) the Nordic model—state support for community-based culture (a model shared by Denmark, Sweden and Norway). Since Zimmer and Toepler’s models (1996) do not fit the reality of cultural policies in Southern Europe, Rubio Arostegui and Rius-Ulldemolins (2020) have added a new cultural policy model that reflects the common characteristics of the Southern European countries (i.e. Portugal, Spain, Italy and Greece). According to the authors, these countries share several distinctive characteristics that allow them to be compared, including their peripheral location in Southern Europe, the fact they are nation states of comparable sizes, their status as EU members for at least thirty years, their democratisation in the eighties—except for Italy—and the different impacts the economic crisis has had on them. Furthermore, all three have developed, albeit with different objectives, instruments and budgets, a specific cultural policy since the 1980s. The Southern European model is characterised by the fundamental weight given to culture, particularly to inherited patrimony. Southern European countries have developed a highly decentralised model with a prominent role at the local level of administrations and public agencies specialised in culture. This has led to remarkable cultural dynamism and innovation, linking with the international tendency towards creative cities and joining European culture programs, such as the European Capital of Culture. However, Southern European cultural policies still remain significantly weaker, both institutionally and in terms of their budgets, than those in the rest of the European continent. Although Southern European countries are among the richest countries in terms of cultural heritage, the limits and weaknesses of their cultural policies—exacerbated by the global economic crisis in 2008—have led to significant reductions in cultural expenditure and inefficiency in public culture actions. The cultural policies of Southern Europe are still far from reaching the power of dissemination and promotion of culture that belongs to the models of Central and Northern Europe (Rubio Arostegui & Rius-Ulldemolins, 2020).

The emergence of “participatory” digital platforms has caused major changes in CCIs in the last decade as new forms of online cultural participation (e.g. crowdfunding sites) have appeared (Cicchiello et al., 2022). In the face of the establishment of crowdfunding as a valid alternative or complementary mode of funding for cultural and creative projects and ventures, cultural policies can play a key role in helping legitimise this form of private financing and foster cultural participation through digital means (Casemajor et al., 2021). Prior studies have used the institutional context to analyse what makes a crowdfunding campaign successful (e.g. Di Pietro & Butticè, 2020). However, the question of how the institutional context may influence the adoption of crowdfunding itself has not been explored. Here, we draw on welfare state theory (Zimmer & Toepler, 1996) to explain how different cultural policies can influence the adoption of reward-based crowdfunding in CCIs. This distinction is important to guide cultural and creative actors in choosing to use crowdfunding as an alternative or in addition to traditional forms of fundraising.

National cultural policy frameworks are generally defined by the Ministry of Culture, which is the government ministry charged with the preservation, promotion and dissemination of all forms of art and culture. Overall, the Ministry of Culture is one of the most important promoters of a country’s official culture. It sets the direction for government policy across the cultural sector by funding and organising cultural events and projects, as well as fostering cultural and intellectual interaction with the outside world. Over the last few years, researchers have underlined the vital role played by the Ministry of Culture as the official sponsor of cultural affairs and activities in funding, supporting and maintaining many cultural establishments, especially in countries less aware of the problems of safeguarding, conserving and restoring cultural heritage. For example, Al-Saleh (2016) shows how the Ministry of Culture, despite being a product of the political regime, has successfully promoted cultural development within Syria by producing a large amount of cultural resources and providing Syrian intellectuals with safe artistic places in which to express themselves freely beyond political orientations. According to Von Maltzahn (2018), the presence of the Ministry of Culture is essential to support culture as long as it does not interfere with the artistic freedom of Lebanese artists and cultural players. In some European countries (e.g. Italy, Spain, France, Portugal), the system of cultural policy is strongly centralised and managed by the central government and the Ministry of Culture with direct responsibility to generate a legitimate pattern of behaviour and cultural practice, which radiates throughout the national territory (Rius-Ulldemolins et al., 2021). The adoption of a centralised system in cultural policy, in which the national culture is kept and arbitrated by a small circle of people and institutions, can create a gap between the centre where the institutions are located (usually the capital) and the peripheries. This is especially true for federal states and for states that are made up of historical autonomous communities with a high degree of autonomy and with cultural or national minorities. In this context, the adoption of the decentralised model in cultural policy has constituted a growing trend. Partly due to the growing processes of economic globalisation and regional integration, cultural policies of other European countries (e.g. Belgium, Germany, the UK) experienced a decentralisation process in the late twentieth century, in which regional and local governments gained new centrality, while the Minister and the Ministry of Culture lost power. In decentralised systems, the leading funders of culture are local and regional governments, while the central government contributes only a minor part. In Europe, the processes of decentralisation of cultural policies have taken different forms. In Belgium, for example, cultural competencies are decentralised at the level of Communities. In Germany, whose federal structure includes four levels of government (central state, Länder, districts and municipalities), cultural competencies are fully decentralised at the Länder level. In this paper, we investigate whether the adoption of crowdfunding in CCIs is influenced by the presence of a central ministry of culture rather than by a decentralised system in which culture is led by local and regional governments. The Ministry of Culture is responsible for the development of national cultural policy and the achievement of its objectives through legal and financial instruments. As happened in Italy in 2015,^6^ the Ministry of Culture could sponsor interventions in the government to encourage the adoption of crowdfunding in the cultural and creative fields. Therefore, there is good reason to expect countries with a central ministry to be more open to the use of crowdfunding than countries with a decentralised system.

The CCIs are often financially supported by grants launched at the regional, national or European level. For example, to financially support the creative, cultural and audio-visual sectors, the European Union launched the Creative Europe Programme on 1 January 2014. The programme, completed in 2020 and followed by the new Creative Europe programme 2021–2027, was open to cultural and creative organisations from EU Member States, as well as non-EU countries, and offered a budget of €1.47 billion. In addition to offering the benefit of the funding, grants play an important role in certifying to investors the quality of the firm or project (Islam et al., 2018). Applying for a government grant is a highly competitive, time-consuming and costly process; the questions are subjected to rigorous evaluation by a jury of experts (Lerner, 2000). This discourages low-quality firms from participating, as only the best will be able to obtain the grant. Furthermore, by alleviating information asymmetries, grants make it easier for firms to obtain additional resources (Kleinert et al., 2020). In this sense, it is reasonable to assume that receiving funding through government support programs can boost crowdfunding adoption, especially in industries that face difficulties in raising funding, such as CCIs.

In the light of the above, we hypothesise the following:

#### **H2:**

 National cultural policies (i.e. the welfare state model adopted, the presence of a central ministry with cultural competence and the EU grants received) affect the adoption of reward-based crowdfunding in CCIs.

To isolate the “pure” effect of cultural dimensions from the additional effect related to cultural policies, we analyse whether the effect of national cultural dimensions on crowdfunding adoption is moderated by EU grants.

As scholars have reported, firms receiving a government grant are supposed to be more likely to attract additional funding (Kleinert et al., 2020). The central idea is that grants can play an important role in signalling firm quality and building the legitimacy they need to establish ties and obtain funding from key resource providers. For example, Islam et al. (2018) show that start-ups that win prestigious government research grants are 12% more likely to acquire subsequent venture capital (VC) funding. The role of government grants in CCIs has been receiving increasing attention from both the academic and policy spheres (Bakhshi et al., 2015).

As for any other industry, crowdfunding adoption in CCIs may depend on the availability of other traditional funding mechanisms, such as grants. The reality of new business models in the CCIs is characterised by the hybridisation of finance, and the cultural and creative entrepreneurs are responsible for the choice of funding sources: bank loans, guaranteed loans by governmental or non‐profit agencies, crowdfunding, grants and subsidies. Although it is difficult to measure the time and cost involved in a crowdfunding campaign versus a traditional grant application, it is clear that a successful crowdfunding campaign requires a significant investment of time and communication efforts to handle hundreds and thousands of supporters. According to extant research, project creators can be discouraged by the amount of work required to address such a large audience, and therefore, they may prefer to apply for grants (where available) rather than adopt crowdfunding (Gerber & Hui, 2013). At the same time, the benefits and the experience itself of a crowdfunding campaign can be much more rewarding than a grant application. Besides raising financial resources, crowdfunding allows creators to raise awareness of their work, establish connections with others, maintain project control, and learn new skills (Gerber & Hui, 2013). Crowdfunding is also an alternative for firms operating in industries where traditional funding is scarce, and it has the potential to bridge the funding gap.

Based on the above arguments, this study addresses the issue of the conditions under which cultural dimensions benefit crowdfunding adoption in CCIs and investigates the moderating effect of EU grants on the relationships between cultural dimensions and crowdfunding adoption. We assume that the effect of cultural dimensions on crowdfunding adoption in CCIs is contingent on the availability of EU grants and, therefore, can be stronger when more (or less) grants are available. Accordingly, we develop the following hypothesis:

#### **H3:**

 EU grants act as a moderator in the relationship between cultural dimensions and crowdfunding adoption in CCIs.

## Research methodology

### Data and sample

**Fig. 2:**
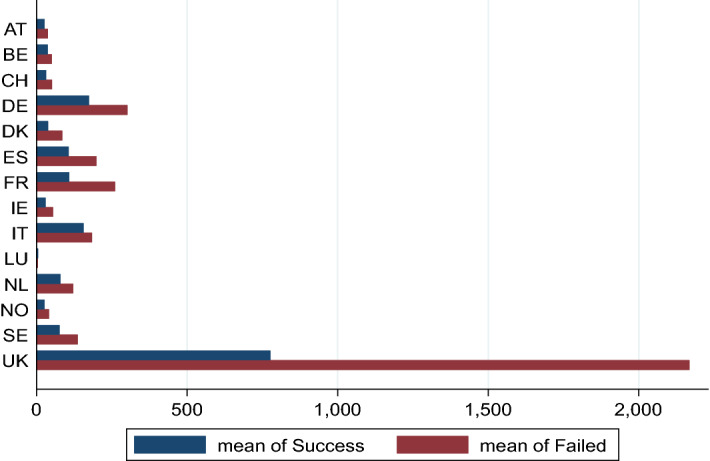
Number of successful and failed projects by country and year. *Note* This figure displays the mean of success and failed of campaigns launched on Kickstarter between 2015 and 2019 in cultural and creative categories in the following 14 European countries: Austria (AT), Belgium (BE), Switzerland (CH), Germany (DE), Denmark (DK), Spain (ES), France (FR), the United Kingdom (UK), Ireland (IE), Italy (IT), Luxembourg (LU), Netherlands (NL), Norway (NO), and Sweden (SE)

This paper investigates the impact of cultural dimensions and policies on the adoption of reward-based crowdfunding in CCIs in 12 different European countries during the 2015–2019 period. As the goal of this paper is to provide the broadest possible perspective of the role of culture dimensions and policies on crowdfunding, the data are extracted from Kickstarter, the largest and dominant reward-based crowdfunding platform based in the UK. The choice to test our hypotheses in this context makes our results comparable with the findings in the literature, as several studies use these data (Mollick, 2014; Yu et al., 2017; Cox et al., 2018; Lazzaro & Noonan, 2020). We use the universe of creative and cultural projects created on Kickstarter between 2015 and 2019.^7^ Kickstarter works according to the traditional “all-or-nothing” model (Cumming et al., 2020a, b); thus, a project is considered successful or funded only if at least 100% of the funding goal is reached within the specified period, which is generally 30–60 days. However, all campaigns (successful and unsuccessful) are retrieved because we aim to explore crowdfunding adoption regardless of whether the funding goal is reached. Specifically, the data used throughout the analysis period originate from several different sources. First, we obtained information on campaigns from the Kickstarter platform’s website. We hand-collected the following types of information: (1) information on the country of the founders, filtering only the projects launched in the EU between 2015 and 2019, and (2) information about the categories in which the campaigns are listed. In our sample, the projects listed in the Comics category have the highest success rate (87%), followed by Design (86%) and Games, while the projects listed in the Food and Technology category report the highest failure rate. The summary of these data for all official categories can be found in Appendix 1. The platform lists the projects in 15 official categories, of which 13 belonged to the cultural and creative sectors according to the European Parliament policy (EU, 2016/2072 (INI)). We dropped all the projects listed in the Food and Technology categories. The final sample is made up of 27,880 campaigns,^8^ of which 12,354 (46%) were successful in reaching their fundraising goal and 6134 (23%) are still ongoing. Figure 2 shows the number of successful and failed projects by country and year.

Second, we matched these data with country-level information from several publicly available databases. Specifically, we extracted the variables related to cultural dimensions from the Hofstede dataset (Hofstede, 1991, 2001). To investigate cultural policy, we considered the Creative Europe Programme,^9^ a specific framework enacted by the European Commission to support the culture and audio-visual sectors. Then, we complemented these data with the World Development Indicators published by the World Bank dataset, which contains time-series information updated yearly for developed and developing economies. The data on information communication and technology (ICT), as well as the data concerning the institutional environment, are obtained from this dataset. Third, we extracted data on companies active in the cultural and creative sectors from the Eurostat database, the statistical office of the European Union. Finally, we obtained information on banking market concentration and national cultural policy from the European Central Bank (ECB) and the European Commission, respectively. Our final database contains information on reward-based crowdfunding in 14 European countries. However, because of missing data on some specific dimensions, our econometric models are based on a smaller sample of 12 countries.

### Variables

**Fig. 3:**
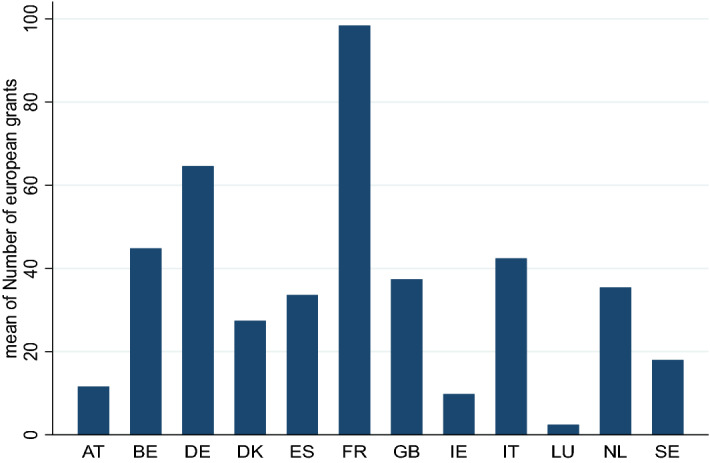
Number of EU grants awarded under the Creative Europe Programme by country (2015–2019). Source: Authors’ elaboration based on Creative Europe Programme data. *Note* This figure reports the number of EU grants by 12 EU countries involved in our dataset over the period 2015–2019

To assess the linkage between reward-based crowdfunding and the national cultural, institutional and economic environment, we use as dependent variable “*Crowdfunding adoption*”, measured by the total number of successful and unsuccessful campaigns created in the cultural and creative categories in a particular country in a given year. This measure has been commonly adopted to explore the development of crowdfunding in the literature as an alternative source of funds (e.g. Block et al., 2018). We also filter the number of successful cultural and creative projects which serves as the dependent variable in our robustness check. The independent variables of interest are the national cultural dimensions which collectively portray the impact of the culture ingrained in society on the values of the members of that society. We consider the six cultural dimensions of Hofstede’s national culture (1991, 2001). Specifically, we include an index of *individualism* versus collectivism, which captures the degree at which people gives priority to their personal goals over in-group goals. A measure of *uncertainty avoidance* is to explore how much people feel uncomfortable towards uncertain and ambiguous events. We consider a variable to capturing *power distance* dimensions expressing the degree to which the less powerful members of a society accept and expect that power is distributed unequally. We insert a variable for *masculinity* denoting the striving of society for achievement, material rewards, heroism and recognition rather than for cooperation, modesty, caring for the weak and quality of life. Finally, we consider a variable for *indulgence*, that is, the importance in society to allowing freedom, leisure, happiness, free gratification and a measure for the *country’s long-term versus short-term orientations* stands for the preference to maintain traditions and norms by viewing the change with distrustful. In line with previous studies (Di Pietro & Butticè, 2020), we expect these cultural characteristics to influence crowdfunding activities in the CCIs. Three variables are used as proxies for national cultural policies. First, we adopt the traditional Welfare State framework by Zimmer and Toepler (1996) in a renewed version proposed by Rubio Arostegui and Rius-Ulldemolins (2020). We create a dummy variable for each model of cultural policy (i.e. liberal, central-European, Nordic and south Europe). The second policy variable attempts to capture whether the management of the cultural system is centralised or decentralised at the local level. Thus, we create the dichotomous variable “*central ministry*”, denoting 1 for centralised systems (i.e. countries having a central ministry with cultural competence) and 0 otherwise, following the report on culture by the European Statistical System Network (ESSnet). As the third proxy for national cultural policies, we consider Europe’s involvement in supporting creative and cultural firms. Specifically, we include a variable representing the total number of EU grants awarded under the *Creative Europe Programme* in a specific country in a given year. Also, we use the EU grants variable as a moderator between cultural dimensions and the adoption of Crowdfunding. Figure 3 displays the number of EU grants by country.

Following Johnstone et al. (2012), we consider differences in the environmental innovation^10^ by including as a proxy of country’s innovativeness a variable measuring the number of patent applications at the European Patent Office (EPO) registered by specific country in a given year. Moreover, we use the ease of doing business index (from the World Bank) in the national market that captures how much the regulatory environment is more conducive to the starting new business (Abu Amuna et al*.*, 2017). The index ranks economies on their ease of doing business from 1 to 190. A high index means that the regulatory environment fosters the attitude towards entrepreneurial. To assess the factors that can boost the adoption of crowdfunding services in each country, we use a set of three proxies, represented by the following variables: the percentage of the economically active population in each country in a given year as a proxy of the dimensions of national market (Dushnitsky et al., 2016); the cultural employment rate measured by the percentage of the population engaged in cultural and creative activities, according to the classification of economic activities in the European Commission (i.e. NACE), within the total employed population. This variable serves as a proxy of the degree of vitality and dynamism of the cultural sectors as well as its ability to increase the national welfare. Also, we use a vector of information and communication technology (ICT) variables (i.e. the percentage of households’ internet usage), and we record the number of internet secure serves in a country in a specific year as a proxy of the level of trust in internet adoption. Higher values of these variables can result in the potential applications for crowdfunding services. Finally, we also include various economic factors at the country level serving as control variables in the empirical model. We consider the following variables: (1) per capita GDP to control for available average wealth at country level; (2) The national banking market concentration measured by the Herfindahl–Hirschman index (HHI) to explore the degree of the involvement of the banking industry in a country. In line with previous studies (Colombo et al., 2020; Rau, 2020), we expect that country with a lower banking market concentration presents a highly developed crowdfunding market. Moreover, we take into account the general government expenditure on cultural services to control for the state engagement in promoting and supporting cultural and creative industries.

Data sources and variables are presented in Table 1.

**Table 1:** Note on variables and data source

Variable	Description	Data sources
Dependent variable		
Crowdfunding adoption	Total number of crowdfunding campaigns created in creative and cultural categories in a particular country in a given year	Kickstarter website
Explanatory variables		
Cultural dimensions	The six dimensions of Hofstede’s cultural values: individualism, power distance, masculinity, uncertainty avoidance, long term orientation and indulgence	Hofstede dataset
EU grants	Total number of EU grants awarded in a particular country in a given year	European Commission
Welfare state model	Categorical variable based on the Welfare state model. It takes value 1 for the Liberal model (the UK, Ireland); value 2 for the Central European model (Austria, Belgium, Germany, France, Netherlands and Luxembourg); value 3 for the Nordic model (Denmark and Sweden); and value 4 for the Southern-European model (Spain and Italy)	Zimmer and Toepler (1996), Rubio Arostegui and Rius-Ulldemolins (2020)
Central Ministry	Dummy variable equals to 1 if there is a Central ministry with cultural competence in a given country, 0 otherwise	European Statistical System Network on Culture (ESSnet-Culture)
Crowdfunding performance	The ratio of pledge over goal	Kickstarter
GDP per capita	Per capita GDP (annual %)	Eurostat
Cultural employment	The number of persons employed having either a cultural profession, or working in the cultural sector (Thousand)	Eurostat
Household level of internet	Individuals using the internet at home (% of population)	World Bank data
Trust in Internet operation	Number of secure internet servers (Thousand)	World Bank data
Herfindahl index for credit institutions	Index refers to the concentration of banking business. A high value means a higher concentration of the banking sector in a given country	European Central Bank (ECB)
European Patent applications	The total number of European patent applications in a given country in a specific year	European patent office (EPO)
Ease of doing new business	Countries are ranked on their ease of doing business, from 1 to 190. A high number means the regulatory environment is more favourable to the starting of a local firm	World Bank data
Active population	The number of population 15–64 aged in a given country in a specific year	World Bank data
Internet banking	Individuals using the internet banking (% of population)	World Bank data

### Methods

To investigate the relationship between reward-based crowdfunding in the CCIs and national cultural dimensions and policies, we employ Poisson regression. We use Poisson regression because of the general characteristics of the dependent variable in this study (i.e. count variable). Poisson probabilities are used to model the number of occurrences of an event (Greene, 2003; Cameron and Trivedi, 2013) and are widely used in entrepreneurship research (Haeussler et al*.*, 2012). However, given the panel structure of our data, following Wooldridge (2019) we estimate Poisson regression with correlated random effects (CRE).^11^ We adopt this model to explore time-variant and time-invariant factors affecting the response variable by addressing and modelling endogeneity and heterogeneity issues. The model is specified as follows:

$$E\left[ {y_{{i1}} |x_{{i1}} , \ldots ,x_{{iT}} ,\,\alpha _{i} } \right] = {\text{exp}}(x_{{it}}^{\prime } \beta + \overline{{x_{i}^{\prime } \lambda }} + \varepsilon _{i} )$$

In this model, $\overline{{x_{i}^{{\prime}} \lambda }}$ represents the vector of time averages proposed originally by Mundlak (1978) and afterwards relaxed by Chamberlain (1984). The term correlated random effects denoting situations where we model the relationship between $y_{i1}$ and $x_{i1}$. Correlated random effects (Wooldridge, 2010) and hybrid models (Allison, 2014) can estimate within effects in random-effects models. These models, therefore, are useful extensions to the standard random effects (RE) and fixed effects (FE) approaches (Schunck, 2013). This approach allows us the inclusion of time-constant variables and at the same time provides FE estimates of time-varying variables. It thus combines the advantages of FE and RE models and increases flexibility in model set-up. It extends traditional random parameters modelling in which the sources of heterogeneity are assumed to be independent (Fountas et al., 2018; Mannering et al., 2016). CRE approach allows unobserved heterogeneity to be correlated with observed covariates that are explicitly captured by applying the unrestricted covariance matrix of the random parameters (Fountas et al., 2018). Specifically, the CRE model allows us to include the cluster mean of $x_{it}$ in the RE method. In order to run the CRE model, we generate a panel-unit-specific mean of all time-varying regressors and then we include their average value within time-invariant variables. Hence, the coefficients for time-variant variables are estimated with the RE model, whereas the coefficients for time-varying predictors are based on FE estimation.

In examining the relationship between the adoption of reward-based crowdfunding in the CCIs and national cultural dimensions and policies, we first present a set of analyses where we associate the demand of crowdfunding with the national cultural dimensions, controlling for country-specific characteristics. We separately test three indicators of internet penetration that are commonly used to measure the development of crowdfunding (e.g. household level of the internet at home, Internet banking and secure servers). We add environmental innovation (patent applications), cultural employment, and as country-level controls the level of GDP per capita and the banking market concentration. In a subsequent analysis, we test the effect of policy variables on the response variable. Specifically, (1) we add the management of cultural system at a country level and categorical variable denoting the welfare state; (2) we replicate our models by including the number of EU grants and its squared term to capture the nonlinear relationship with the response variable; and (3) we include the interaction terms to test the moderation effects of policy culture between cultural dimensions and crowdfunding adoption. Finally, we re-run our econometric model with the number of successful Kickstarter projects as a dependent variable to test the robustness of our results.

## Results

As reported in Table 2, the average level of public cultural expenditure (as a percentage of GDP) is equal to 45%, and the average number of cultural and creative projects on Kickstarter is equal to 468.4. As shown in Table 3, France represents the largest cultural expenditure per capita (56.3%), followed by Belgium (52.5%) and Denmark (51.8%). The average amount issued under the Creative Europe Programme on the GDP per capita is the highest in France, again followed by Belgium.

**Table 2:** Summary statistics

	Mean	SD	Min	Max
Crowdfunding adoption	468.4	851.3	7	4198
Individualism	69.9	10.2	51	89
Power distance	39.7	16.9	11	68
Long term orientation	57.7	16.5	24.4	82.9
Indulgence	57.2	13.8	29.7	77.7
Masculinity	47.7	23.7	5	79
Uncertainty avoidance	60.1	23.6	23	94
Performance of crowd	4.5	2.6	0.4	10.8
GDP growth	2.1	3.2	− 0.4	24
Cultural employment	562.9	536.2	12.4	1677
Environmental innovation	5510.7	6726.1	420	26,715
Herfindahl index	392.1	307.2	1.1	998
Active population	14,950.6	13,779.5	274	42,427
Ease of doing business	77.2	5.8	57	85.3
Central ministry	0.7	0.5	0	1
Internet banking	63.4	16.9	28	91
EU grants	35.5	29.4	2	153
Secure servers	29,604.8	44,015.2	628	277,133
Government expenditure	45.6	7.6	24.3	56.8
Households level of internet	0.9	0.1	0.7	0.9
Cultural policy	2.3	0.9	1	4

**Table 3:** Public and private cultural expenditure by country

Country	Government expenditure on culture (% of GDP)	Volume of EU grants (in millions €)	Number of Kickstarter cultural and creative project
Austria	49.5	97.17	69
Belgium	52.5	318.95	93
Germany	44.4	385.01	519.2
Denmark	51.8	96.11	134
Spain	42	239.39	337.2
France	56.3	773.06	399
UK	41.2	258.84	3145.2
Ireland	26.5	23.02	91.8
Italy	48.8	340.16	371
Luxembourg	41.6	7.99	9.4
Netherlands	42.8	180.61	221.2
Sweden	49.6	76.74	230.4

This table reports public cultural expenditure, such as Government expenditure and volume of EU grants, and private cultural investment representing from Kickstarter cultural and creative projects

We report the results of our baseline models in Table 4. Models (1)–(4) show the effect of country-level cultural dimensions on the number of cultural and creative projects by including control variables. Overall, national cultural dimensions significantly affect the demand for cultural and creative crowdfunding at the 1% level. In line with previous studies (e.g. Di Pietro & Butticè, 2020), Model 1 shows a positive and statistically significant association between individualistic countries and the dependent variable. This suggests that the adoption of reward-based crowdfunding for cultural and creative activities is broader in countries with weak ties between individuals, characterised by the absence of cohesive groups. We find that power distance is negatively associated with crowdfunding demand. This suggests that cultural enterprises that value equal opportunities for all individuals are more encouraged to apply for innovative funding. We also observe a positive and statistically significant relationship between uncertainty avoidance and the number of campaigns.

**Table 4:** Correlated Random effects (CRE) approach in Poisson regression

Dependent variable	Crowdfunding adoption			
	(1) CRE	(2) CRE	(3) CRE	(4) CRE
Individualism	0.044*** (0.009)	0.084*** (0.010)	0.093*** (0.024)	0.070*** (0.020)
Power distance	− 0.028*** (0.009)	− 0.055*** (0.010)	− 0.050** (0.022)	− 0.061*** (0.019)
Uncertainty avoidance	0.027*** (0.010)	0.049*** (0.009)	0.062*** (0.023)	0.045** (0.018)
Long-term orientation	− 0.016** (0.007)	− 0.027*** (0.005)	− 0.035*** (0.013)	0.010 (0.019)
Masculinity	− 0.028*** (0.004)	− 0.029*** (0.004)	− 0.035** (0.016)	− 0.032*** (0.008)
Indulgence	0.049*** (0.007)	0.047*** (0.006)	0.076** (0.033)	0.033*** (0.010)
Households level of internet	4.374*** (0.589)			
Cultural employment	0.002*** (0.001)			
GDP growth	0.005 (0.005)	0.017*** (0.005)	0.013** (0.005)	0.019*** (0.005)
/lnalpha	− 3.663*** (0.421)	− 3.800*** (0.430)	− 2.400*** (0.426)	− 2.840*** (0.448)
Secure servers		0.198*** (0.033)		
Active population		− 0.001*** (0.000)	− 0.000*** (0.000)	− 0.000*** (0.000)
Internet banking			− 0.013*** (0.003)	
Herfindahl index			− 0.001*** (0.000)	− 0.000*** (0.000)
Environmental innovation				0.000*** (0.000)
Year fixed effects	Yes	Yes	Yes	Yes
/lnalpha	− 3.663*** (0.421)	− 3.800*** (0.430)	− 2.400*** (0.426)	− 2.840*** (0.448)
Constant	13.461*** (1.851)	4.295*** (1.122)	− 3.139 (2.181)	− 3.548** (1.642)
Likelihood-ratio test Chibar2	415.631	583.361	244.291	109.801
Prob > Chibar2	0.000	0.000	0.000	0.000
Wald test Chi2	2065.281	1631.921	1558.121	1646.831
Prob > Chi2	0.000	0.000	0.000	0.000
Pseudo-Hausman test Chi2	86.991	110.191	29.251	87.951
Prob > Chi2	0.000	0.000	0.000	0.000

Standard errors in parentheses ***p < 0.01; **p < 0.05; *p < 0.1

These results are estimated with correlated random effects (CRE) approach. Time-varying variables are calculated with FE estimations, while time-constant variable with RE estimations. Likelihood-ratio test, Wald test and pseudo-Hausman test are reported in the table for all specifications. In specific, pseudo-Hausman test is a renewed version of the Hausman test created by Wooldridge (2002) and was run as the goodness of fit

In line with Cumming et al. (2017), we expected a negative relationship between cultures with high levels of uncertainty and engagement in crowdfunding campaigns. However, we find a positive and significant impact on crowdfunding in terms of reaching the fundraising goal. This result can be explained by the fact that the platform Kickstarter depicts lower levels of uncertainty than do other sites, thereby making reward funding more trustworthy (Cho & Kim, 2017). Additionally, the positive impact of indulgence on reward crowdfunding indicates that cultural and creative firms have a preference for more innovative models of fundraising. This is because in a country where freedom expression, happiness and enjoyment in life are encouraged, the potential failure of a firm to reach fundraising goals is not reputed as shameful, and individuals feel free of constraints. There is a negative association between masculinity and reward crowdfunding. This result confirms previous studies on entrepreneurship asserting that in masculine societies, there is a stronger attitude towards enterprise (Hayton et al., 2002), which in turn can result in a higher rate of applications for equity crowdfunding due to the stronger material-reward orientation. Unlike previous studies (e.g. Di Pietro & Butticè, 2020), our results show a negative association between long-term orientation and the application of crowdfunding. Thus, cultural and creative firms operating in a country where there is a preference for long-term orientation are intrinsically less prone to launch a reward-based crowdfunding campaign. Overall, our results are in line with Hofstede’s cultural dimensions theory (Hofstede, 2001), according to which a society’s culture affects the values of its members and their behaviours, promoting or discouraging the diffusion of new phenomena, such as crowdfunding. In relation to the variables used as proxies for the demand for crowdfunding, in Model 1, we can observe that increases in the level of internet access at home and in the number of people employed in cultural and creative activities increase applications for crowdfunding. These effects are positive and significant at the 1% level. In Model 2, cultural dimensions still affect the adoption of cultural and creative crowdfunding at the 1% significance level. We substitute the previous demand factors with population density and the level of general trust in internet applications. Relying on the theory of the diffusion of innovations (DIT) (Rogers, 2014),^12^ our results confirm that countries with a high reliability of e-commerce have the largest number of crowdfunding projects (De Leon & Mora, 2017). This result seems to be consistent with the idea that internet protection through a predetermined protocol reduces the level of uncertainty associated with crowdfunding activity. Moreover, population density and the growth of GDP per capita have, respectively, a negative and a positive impact on the response variable. In Model 3, we control for banking market concentration and the extent to which the internet is used for online banking transactions to explore the relationship between traditional finance and cultural and creative firms. We find that the Herfindahl index and internet banking are negatively associated with crowdfunding adoption. These results suggest that the development of cultural and creative crowdfunding is more developed in countries where the banking market concentration is lower as a consequence of crowdfunding’s substitution effect. Finally, in Model 4, we add the variables related to environmental innovation. The coefficient of European patent applications is positively and significantly correlated with the number of campaigns (*p* < 0.01). This result suggests that there is a higher demand for crowdfunding in cultural and creative sectors in countries characterised by innovative start-ups.

Table 5 presents the results of the regression testing the effect of cultural policies on reward-based crowdfunding demand, as well as the moderating effect of EU grants on the relationship between crowdfunding and cultural dimensions. In Model 1, we find a positive coefficient and statistically significant empirical evidence that the presence of a central ministry with cultural competence influences the response variable. This result suggests that countries where the management of the cultural system is centralised are more likely to adopt alternative funding. The coefficient performance of crowdfunding is positively and statistically significant at the 1% level, consistent with previous studies on the relationship between crowdfunding adoption and project success (Lewis et al., 2020). In Model 2, we include the welfare state variables in our empirical model. The results indicate that cultural and creative firms in a cultural policy system based on the liberal welfare state model, characterised by limited government interference, market orientation, privatisation and a focus on self-responsibility, as well as the Southern European welfare state model, based on a weak and inefficient state with weak and limited cultural policies, are more likely to adopt crowdfunding than the reference group (e.g. the Nordic model). Therefore, cultural and creative firms in the UK, Ireland, Spain and Italy are more prone to use alternative sources of financing, such as crowdfunding, to support their growth and development than those in Denmark and Sweden. Therefore, countries in the liberal and Southern European welfare models are the principal applicants of crowdfunding. These results are in line with welfare state theory (Zimmer & Toepler, 1996), according to which the liberal countries model is underpinned by a capitalist economy that encourages creative destruction and laws that make it easy for companies to implement transformative business models. The results also indicate that countries with a central ministry of culture are still positively associated with the adoption of crowdfunding. In Model 3, we explore the relationship between cultural policies and crowdfunding by considering the number of EU grants awarded to support creative and cultural firms. We also include the cultural dimension variables to test how EU grants affect the demand for crowdfunding by holding constant national cultural dimensions. The coefficient for EU grants is positively and significantly correlated with the adoption of crowdfunding, controlling for general government expenditure on culture. However, the squared term of the EU grants is negatively associated with the response variable. This result shows that there is a nonlinear relationship between public support for cultural sectors and crowdfunding investment. This suggests that if European government support goes beyond a certain threshold, it could make it unnecessary to resort to alternative sources of finance, discouraging crowdfunding adoption.

**Table 5:** Correlated random effects (CRE) approach in Poisson regression

Dependent variable	Crowdfunding adoption			
	(1)	(2)	(3)	(4)
	CRE	CRE	CRE	CRE
Central ministry	0.560** (0.250)	1.257** (0.512)		
Liberal policy-cultural model		1.050* (0.557)		
Central policy-cultural model		0.0173 (0.259)		
South policy-cultural model		1.991*** (0.470)		
Performance of crowdfunding	0.049*** (0.008)	0.0495*** (0.008)		
EU grants			0.006*** (0.001)	0.033*** (0.010)
(EU grants)^2^			− 1.45e−05* (8.24e−06)	− 2.37e−05* (1.41e−05)
Government expenditure			0.029* (0.016)	
Uncertainty avoidance				0.064*** (0.018)
Individualism				0.095*** (0.020)
Long-term orientation				− 0.048*** (0.011)
Power distance				− 0.038** (0.016)
Masculinity				− 0.023*** (0.007)
Indulgence				0.057*** (0.013)
EU grants * uncertainty				− 0.000 (0.000)
EU grants * individualism				− 0.000*** (0.000)
EU grants * long term				0.000 (9.20e−05)
EU grants * power				0.001* (0.000)
EU grants * masculinity				9.76e−05 (8.25e−05)
EU grants * indulgence				− 0.003*** (0.000)
Cultural employment	0.001* (0.000)	0.001* (0.000)	0.002*** (0.000)	
Active population				− 0.000*** (5.97e−05)
Households level of internet	4.527*** (0.662)	4.523*** (0.662)	3.610*** (0.678)	
Gdp growth	0.016*** (0.005)	0.016*** (0.005)	0.003 (0.005)	
Herfindahl index	− 0.000*** (5.61e−05)	− 0.000*** (5.61e−05)		
Ease of doing business	0.053*** (0.017)	0.053*** (0.0173)	0.031* (0.017)	
/lnalpha	− 2.082*** (0.411)	− 2.350*** (0.410)	− 1.856*** (0.401)	− 2.340*** (0.433)
Year fixed effects	Yes	Yes	Yes	Yes
Constant	− 2.430 (2.625)	− 3.483 (4.368)	8.414 (5.245)	− 4.461*** (1.656)
Likelihood-ratio test Chibar2	959.46	801.81	3363.881	187.511
Prob > Chibar2	0.000	0.000	0.000	0.000
Wald test Chi2	1561.951	1618.461	1573.231	1623.781
Prob > Chi2	0.000	0.000	0.000	0.000
Pseudo-Hausman test Chi2	25.511	18.831	17.691	40.211
Prob > Chi2	0.000	0.004	0.007	0.000

Standard errors in parentheses ***p < 0.01; **p < 0.05; *p < 0.1

These results are estimated with Correlated random effects (CRE) approach. Time-varying variables are calculated with FE estimations, while time-constant variable with RE estimations. These results are estimated with Correlated random effects (CRE) approach. Time-varying variables are calculated with FE estimations, while time-constant variable with RE estimations. Likelihood-ratio test, Wald test and pseudo-Hausman test are reported in the table for all specifications. In specific, pseudo-Hausman test is a renewed version of the Hausman test created by Wooldridge (2002) and was run as the goodness-fit

We also test the effect of the legal environment on the adoption of crowdfunding in cultural and creative firms. In Models (1)–(3), the ease of doing business index positively impacts the response variable, suggesting that the development of crowdfunding is encouraged in a favourable legal environment. This result is also confirmed by previous studies on the relationship between the development of crowdfunding and the formal institutional context (Di Pietro & Butticè, 2020). Finally, the last column explores the moderation effect between EU grants and national cultural dimensions on the development of reward crowdfunding in the cultural and creative sectors across countries. To examine this statistical interaction, we used the six dimensions of Hofstede’s cultural values (namely, uncertainty avoidance, individualism, power distance, long-term orientation, masculinity and indulgence). The multiplicative product of each cultural variable and the moderating variable EU grants, a proxy of European cultural policy, is used as a predictor to identify the moderation effect. As reported in Model 4, significant moderating effects are observed for three cultural dimensions over the total dimensions, so H_3_ is partially confirmed. Individualism as an interaction term (EU grants*Individualism) significantly affects crowdfunding applications at the level of 1%, as does Indulgence. The relationship between national culture and crowdfunding is also moderated by Power distance at the 10% level of significance. European financial support negatively moderates the relationship between crowdfunding demand and Individualism and Indulgence, while the interaction with Power distance positively moderates the effect on the response variable. The interaction between EU grants and long-term orientation, masculinity and uncertainty avoidance does not have a significant or moderating effect on crowdfunding adoption.

### Robustness check

To confirm and make our results more reliable, we conduct a further check by replicating our econometrics models with the number of successful campaigns in cultural and creative sectors as the dependent variable. Since the new response variable has the same characteristics as the previous one (e.g. a count variable that takes only integer values), we prefer to replicate the investigation of the propensity to adopt reward-based crowdfunding by applying correlated random effects in Poisson regression. Thus, we estimate the same models adopted in Table 4 using the new dependent variable and report the results in Tables 6 and 7.

**Table 6:** Robustness check

Dependent variable	Crowdfunding adoption			
	(1)	(2)	(3)	(4)
	CRE	CRE	CRE	CRE
Individualism	0.035*** (0.008)	0.065*** (0.011)	0.042*** (0.015)	0.064*** (0.019)
Powerdistance	− 0.015* (0.009)	− 0.032*** (0.0121)	− 0.028* (0.014)	− 0.022 (0.018)
Long-term orientation	− 0.023*** (0.006)	− 0.030*** (0.006)	0.004 (0.013)	− 0.032*** (0.010)
Uncertainty avoidance	0.020** (0.009)	0.033*** (0.011)	0.018 (0.014)	0.036* (0.018)
Masculinity	− 0.022*** (0.004)	− 0.020*** (0.005)	− 0.019*** (0.006)	− 0.024** (0.012)
Indulgence	0.054*** (0.006)	0.056*** (0.007)	0.044*** (0.007)	0.082*** (0.026)
Active population		− 0.001* (0.000)	− 0.000*** (0.000)	4.04e−05 (9.55e−05)
Environmental innovation			0.000*** (0.000)	
Herfindahl index			− 0.001*** (0.000)	− 0.000** (0.000)
GDP growth	− 0.009 (0.009)	0.00205 (0.0101)	0.009 (0.009)	− 0.004 (0.009)
/lnalpha	− 4.072*** (0.457)	− 3.671*** (0.462)	− 3.551*** (0.604)	− 2.863*** (0.488)
Cultural employment	0.002*** (0.000)			
Households level of internet	3.644*** (1.060)			
Secure servers		0.408*** (0.0577)		
Internet banking				− 0.033*** (0.005)
Year fixed effects	Yes	Yes	Yes	Yes
/lnalpha	− 4.072*** (0.457)	− 3.671*** (0.462)	− 3.551*** (0.604)	− 2.863*** (0.488)
Constant	9.992*** (1.778)	2.317* (1.227)	− 2.685** (1.266)	− 4.461 (1.656)
Likelihood-ratio test Chibar2	86.381	159.131	8.781	244.291
Prob > Chibar2	0.000	0.000	0.000	0.000
Wald test Chi2	1717.741	1191.151	1429.171	1558.121
Prob > Chi2	0.000	0.000	0.000	0.000
Pseudo-Hausman Test Chi2	47.781	45.571	78.791	29.251
Prob > Chi2	0.000	0.000	0.000	0.000

Standard errors in parentheses ***p < 0.01; **p < 0.05; *p < 0.1

The dependent variable is the number of successful campaigns in cultural and creative categories on Kickstarter in all model. The results are estimated with Correlated random effects (CRE) approach. Time-varying variables are calculated with FE estimations, while time-constant variable with RE estimations

**Table 7:** Robustness check

Dependent variable	Crowdfunding adoption			
	(1)	(2)	(3)	(4)
	CRE	CRE	CRE	CRE
Central ministry	0.558** (0.222)	0.983** (0.422)		
Central policy-cultural model		3.519* (1.909)		
Nordic policy-cultural model		5.812** (2.772)		
Southern policy-cultural model		2.019** (0.958)		
Performance of crowdfunding	0.071*** (0.013)	0.071*** (0.013)	0.097*** (0.013)	
EU grants			0.036*** (0.003)	0.0425 (0.0305)
(EU grants)^2^			− 0.001*** (0.000)	− 0.001*** (0.000)
Government expenditure			0.00967 (0.027)	
Uncertainty avoidance				0.044*** (0.016)
Individualism				0.057*** (0.020)
Power distance				− 0.019 (0.021)
Long term orientation				− 0.043*** (0.013)
Masculinity				− 0.022*** (0.008)
Indulgence				0.074*** (0.008)
EU grants *uncertainty Av				− 0.000 (0.000)
EU grants *individualism				− 0.000 (0.000)
EU grants *power distance				0.000 (0.000)
EU grants *long term				0.000 (0.000)
EU grants *masculinity				0.000*** (0.000)
EU grants *indulgence				− 0.000*** (0.000)
Ease of doing business	0.051* (0.028)	0.051* (0.028)	0.033 (0.030)	
Active population				− 0.000* (0.0002)
Cultural employment	0.000 (0.000)	0.000 (0.000)	0.001 (0.001)	
Households level of internet	3.897*** (1.169)	3.906*** (1.169)	3.885*** (1.184)	
GDP growth	0.008 (0.009)	0.008 (0.009)	0.0180* (0.009)	
Herfindahl index	− 0.000*** (9.17e−05)	− 0.000*** (9.16e−05)		
Year fixed effects	Yes	Yes	Yes	Yes
/lnalpha	− 2.355*** (0.430)	− 2.766*** (0.433)	− 1.905*** (0.408)	− 4.880*** (1.018)
Constant	− 6.617*** (2.361)	− 5.078 (3.691)	− 6.795 (6.466)	− 3.946*** (1.197)
Likelihood-ratio test Chibar2	254.171	178.381	1359.311	195.651
Prob > Chibar2	0.000	0.000	0.000	0.000
Wald test Chi2	1065.821	1161.161	1102.961	1124.591
Prob > Chi2	0.000	0.000	0.000	0.000
Pseudo-Hausman test Chi2	22	19.71	12.78	19.57
Prob > Chi2	0.001	0.003	0.046	0.001

Standard errors in parentheses ***p < 0.01; **p < 0.05; *p < 0.1

The dependent variable is the number of successful campaigns in cultural and creative categories on Kickstarter in all model. The results are estimated with Correlated random effects (CRE) approach. Time-varying variables are calculated with FE estimations, while time-constant variable with RE estimations

As we expected, the robustness tests confirm our main results. The cultural variables still have strong statistical significance for the successful campaigns, and the signs of their coefficients remain unchanged in all specifications, as shown in Table 6. We note that in these estimations, power distance and uncertainty avoidance still have a negative and positive impact on the response variable, respectively, but with weaker explanatory power. However, these findings confirm the association between cultural dimensions and crowdfunding adoption. We also investigate the impact of policy variables on the second dependent variable (i.e. number of successful campaigns), employed in the previous sections in Table 5. The results of the new specifications, reported in Table 7, are in line with previous findings, namely, supporting the possible existence of an association between cultural policy variables and crowdfunding adoption in a country. The only notable exception relates to the moderating effects of European support on the relationship between cultural dimensions and crowdfunding adoption. In these specifications, the interaction term between Masculinity and EU grants is positive and significant, while the interaction term with Indulgence is negative and significant. The other moderating effects have the same impact on this relationship, although with a lower explanatory power than in the specifications in Table 5. Moreover, the evidence on country-level explanatory and controls variables is still strong in terms of both significance and magnitude.

## Discussion and conclusions

Inadequate financing represents a major constraint to the development of cultural and creative businesses in Europe (Collins, 2018). Since these firms are essential for the economic and social growth of countries (Hutter & Throsby, 2008) and are increasingly considered hubs of managerial innovation and experimentation (Lampel & Germain, 2016), crowdfunding can generate great opportunities in European cultural and creative markets (De Voldere & Zeqo, 2017). The aim of this study is to understand to what extent national cultural dimensions and policies can stimulate (or hinder) the adoption of reward-based crowdfunding as a new form of finance for firms in CCIs. Furthermore, we investigate whether EU grants can moderate the relationships between national culture dimensions and crowdfunding adoption.

Our paper presents some major findings and provides some new theoretical insights, as discussed below. First, this study contributes to the emerging literature and knowledge on the country-level determinants of crowdfunding in CCIs (Rykkja et al., 2020) by jointly considering the effect of national cultural dimensions and policies on reward-based crowdfunding activity across European countries. Although prior studies have used context to explore at a microlevel what makes a specific campaign successful (e.g. Josefy et al., 2017), the degree to which the cultural context may influence at a macrolevel the adoption of crowdfunding itself in different environments has not yet been investigated. Our study robustly demonstrates that the demand for cultural and creative crowdfunding can be influenced by national cultural dimensions and policies. In line with previous studies (Di Pietro & Butticè, 2020), our findings reveal that the adoption of crowdfunding is broader in individualistic countries and in societies characterised by higher uncertainty avoidance, indulgence, short-term orientation, and lower levels of discrimination between genders. Furthermore, we find that the liberal welfare state model, characterised by limited government interference, market orientation, privatisation and a focus on self-responsibility, as well as the Southern European welfare model, based on a weak and inefficient state, increase the adoption of crowdfunding in CCIs. The presence of a central ministry with cultural competence also increases the adoption of crowdfunding in CCIs. In addition, the study highlights the existence of a nonlinear relationship between EU grants and the demand for crowdfunding in CCIs: while low or moderate levels of EU grants favour crowdfunding adoption among cultural and creative firms, high levels of grants can discourage its use by these firms. Finally, we identify a partial moderation effect of EU grants on the relationship between national cultural dimensions and crowdfunding adoption in CCIs. While we are careful to keep in mind that there are limitations to the generalisability of our findings outside of this context, our results provide some preliminary practical insights for cultural and creative entrepreneurs and policy-makers for overcoming the funding gap that the prior literature has documented. First, our research has important implications for cultural and creative entrepreneurs, who are financially constrained in traditional entrepreneurial markets (Boeuf et al., 2014; Collins, 2018; Hackett et al., 2000; Landoni et al., 2020; Lazzaro, 2017). Indeed, our results suggest that entrepreneurs in CCIs have higher chances of raising funds through reward crowdfunding in countries characterised by certain cultural dimensions. Thus, from a practical standpoint, it is important that cultural and creative entrepreneurs understand the cultural setting of the country in which they hope to secure funding and adopt tailor-made strategies that take into account the cultural characteristics of the audience of potential supporters. This would enable them to make better choices on the nature of the crowdfunding backers among whom they engage, the manner in which they approach such backers, and the types of products and services for which they might expect support, thereby improving their chances of access to financial resources. For example, when dealing with societies characterised by high uncertainty avoidance, entrepreneurs need to focus more on continuous communication to establish an emotional connection with the audiences of potential backers and encourage them to engage in crowdfunding investment. Similarly, when approaching collectivistic societies, which rely on informal relationships to mitigate information asymmetries and transaction costs (Gould, 1993), entrepreneurs need to increase interactions with potential investors to forge informal relationships and reduce transaction costs. A second implication emerges from this study. Policy-makers have the power to change the environment in which entrepreneurs operate, implementing measures designed to promote a stronger business culture and unleashing the potential of more flexible forms of financing best suited to the needs of firms in the CCIs. Crowdfunding may be a fruitful avenue for cultural and creative entrepreneurs to acquire funding. Understanding how the cultural environment affects crowdfunding adoption is important to support effective policy-making at both the EU and national levels and to define and implement appropriate government policies to encourage the use of crowdfunding. For instance, policy-makers in countries characterised by high levels of uncertainty avoidance could stimulate crowdfunding adoption through the provision of a strong formal institutional framework. Our findings add to the debate on the implementation of appropriate policy frameworks to lower the barriers for cultural and creative actors to access finance and unlock the potential of CCIs. Finally, by showing how national policies significantly affect the demand for cultural and creative crowdfunding, the empirical findings of this study contribute to providing economic validity to the Creative Europe Programme (2014–2020) aimed at helping firms in the CCIs seize the opportunities offered by the Digital Era, including crowdfunding. One of the main objectives of the programme is to increase the global competitiveness and economic potential of the cultural and creative sectors by supporting platforms that increase the visibility and circulation of emerging artists in Europe and beyond and mobilise private investment.

Going forward, the study presents some limitations that should be addressed to provide possible directions for future research. First, similar to much other research in this field, we only analyse data from one crowdfunding platform (i.e. Kickstarter), and this limitation in data availability creates problems for the generalisability of our results. Future studies could expand the experimental setting of our study by including data on creative and cultural campaigns from other platforms and investigate whether our results continue to hold in different contexts, particularly in emerging funding contexts, where culture is increasingly used as a resource for development (Cunningham et al., 2008). Second, in this study, we only considered crowdfunding projects in the CCIs, leaving out the analysis of the democratisation potential of crowdfunding across all other categories of projects listed on the Kickstarter platform. Campaigns in the CCIs tend to differ from those in other categories in many ways, from the relationship between the founder and funders (customers vs. fans) to the nature of the rewards offered (consumer products vs. creative or cultural output) (Tosatto et al., 2019). Therefore, broader studies with regard to the matching between projects in the CCIs and projects in other categories are needed. Third, as we focus on country-level characteristics, future research should investigate factors on the regional level by including additional cultural dimensions not considered in the present study. Finally, this study does not take into consideration the recent socioeconomic crisis due to the COVID-19 pandemic, which has severely affected the cultural and creative industries. Across Europe, almost all cultural activities have been cancelled or postponed indefinitely, with disastrous consequences for the livelihoods of creators and cultural and creative professionals, as well as for the cultural ecosystem as a whole. Future researchers could investigate how the COVID-19 crisis impacts cultural venture activity in the crowdfunding market and what role crowdfunding platforms can play in rebuilding after this crisis and ensuring the survival of the cultural and creative industries.

## Data Availability

Not applicable.

## Appendix 1

See Table 8.

**Table 8:** Summary statistics by category

Categories	Goal (in €)	Pledge/backers	Funded (%)	Failed (%)	Number of backers	Projects (% of total)	Duration (in days)
Art	39,204.6 (878,439)	13.3 (78.2)	0.7 (0.5)	0.3 (0.4)	628 (451.3)	0.10 (0.3)	26.6 (81.8)
Design	30,089.4 (349,053)	79.2 (451.9)	0.9 (0.3)	0.1 (0.3)	704 (431.9)	0.06 (0.2)	71.5 (136.7)
Fashion	25,343.8 (310,275.3)	31.023 (293.3)	0.6 (0.4)	0.3 (0.4)	621 (454.1)	0.08 (0.2)	44.8 (95.1)
Crafts	9835.9 (42,237.81)	6.3 (24.5)	0.5 (0.5)	0.4 (0.4)	574 (449.3)	0.03 (0.16)	26.7 (71.2)
Dance	5878.3 (10,774.02)	6.4 (17.9)	0.6 (0.5)	0.4 (0.4)	639 (448.1)	0.007 (0.07)	22 (29.5)
Film and video	88,778.7 (2,268,130)	16.8 (90.7)	0.7 (0.5)	0.3 (0.4)	616 (452)	0.12 (0.33)	27.5 (74.5)
Technology	77,755.4 (518,576.7)	59.1 (422)	0.3 (0.4)	0.6 (0.4)	577 (462.4)	0.12 (0.33)	49.9 (97.4)
Food	49,753.9 (188,019.9)	17.6 (82.3)	0.3 (0.4)	0.7 (0.4)	541 (470)	0.05 (0.22)	34.3 (85.5)
Journalism	47,769.1 (412,897.2)	11.7 (62.7)	0.3 (0.4)	0.6 (0.4)	463 (470.5)	0.02 (0.13)	24.4 (78.6)
Games	22,461.9 (137,921.8)	193.9 (2224.6)	0.7 (0.4)	0.2 (0.4)	661 (435.8)	0.12 (0.32)	68.9 (141.5)
Comics	6650.4 (33,316.2)	18.4 (73.6)	0.8 (0.3)	0.1 (0.3)	719 (441.4)	0.04 (0.19)	52.5 (112.8)
Music	12,439.8 (63,995.09)	13.7 (154.6)	0.6 (0.4)	0.3 (0.4)	601 (477.9)	0.08 (0.27)	33.01 (92.1)
Photography	10,120.2 (24,501.82)	23.8 (219.1)	0.5 (0.5)	0.4 (0.5)	575 (453.5)	0.04 (0.2)	33.8 (91.1)
Publishing	14,131.3 (57,495.85)	24.2 (137.1)	0.7 (0.4)	0.2 (0.4)	629 (455.9)	0.08 (0.27)	40.5 (94.5)
Theater	137,201.8 (3,210,571)	12.8 (79.1)	0.7 (0.4)	0.3 (0.4)	641 (420.9)	0.03 (0.17)	23 (54.7)

This table displays summaries statistics by category. For each variable we report the average value and standard deviations in parenthesis

## Footnotes

1 I.e., firms that employ fewer than ten employees. Micro-firms are the predominant firm type in the cultural and creative industries.

2 The main types of crowdfunding models include donation-based crowdfunding, reward-based crowdfunding, lending-based crowdfunding and equity-based crowdfunding (Cicchiello, 2019). In donation-based crowdfunding, people donate liberally to social or charitable causes without expecting something in return (Liu et al., 2018). In reward-based crowdfunding, funders support creative entrepreneurial projects in exchange for a nonmonetary reward based on the amount of money they brought into the project (Shneor & Munim, 2019). Lending-based crowdfunding is a feasible alternative to traditional loans, in which funders lend money to consumers or entrepreneurs in return for a certain rate of interest (Kgoroeagira et al., 2019). Finally, in equity-based crowdfunding, investors become shareholders in the funded company through the purchase of a small equity stake (Cicchiello et al., 2021).

3 The countries analysed include Austria, Belgium, Switzerland, Germany, Denmark, Spain, France, the UK, Ireland, Italy, Luxembourg, Netherlands, Norway and Sweden.

4 As defined in Article 2 (point 1) of Regulation (EU) n. 1295/2013 of the European Parliament and of the Council of 11 December 2013 establishing the Creative Europe Programme (2014 to 2020) and repealing Decisions No 1718/2006/EC, No 1855/2006/EC and No 1041/2009/EC.

5 According to the Creative Supply Chains Study (Greater London Authority, 2019), the CCIs contributed £101.5bn of the gross value added (GVA) of the UK economy in 2017; £52.2bn of economic output was generated in London, where the CCIs spent around £40bn within the supply chain even outside the creative sectors. Employment has grown fourfold in creative industries compared to other areas of the economy. In 2017, 267,500 people worked in London’s creative industries, with 203,200 jobs in the creative supply chain—more than in the legal and accounting sectors combined.

6 https://www.crowdfundingbuzz.it/crowdfunding-beni-culturali-e-turismo/.

7 We decided to collect data starting from 2015 because, according to Kickstarter policy, most of the European countries (e.g. Austria, Belgium, Italy, Switzerland and Netherland) were not allowed to launch a campaign before 2015.

8 On Kickstarter’s website, the projects are listed with different status. In this study, we dropped the campaigns with the status “*cancelled by originators*” and “*suspended by the platforms”* because, as a minority, they would bias the estimations.

9 For detailed information, see the website https://eacea.ec.europa.eu/creative-europe_en.

10 Environmental innovations can be defined as specific kinds of innovations consisting of new or modified products and processes aimed at avoiding or reducing environmental impacts and, at the same time, contributing to the technological modernization of the economy (Rennings et al., 2006).

11 CRE approach is applied to the commonly used model, such as Poisson, Tobit and Probit. Papke and Wooldridge (2008) propose a simple CRE method also when the response variable is a fraction or a proportion.

12 This theory seeks to explain how, why, and at what rate new ideas and technology spread through cultures. Rogers defines diffusion as the adoption of an innovation “over time by the given social system”; as a consequence, diffusion processes result in the acceptance or penetration of a new idea, behaviour or physical innovation.
